# Pubertal mediators of early life stress and age on adolescent alcohol initiation: Analysis by sex

**DOI:** 10.1016/j.psyneuen.2025.107559

**Published:** 2025-07-20

**Authors:** Alexandra Donovan, Shervin Assari, Christine Grella, Magda Shaheen, Linda Richter, Theodore C. Friedman

**Affiliations:** a Department of Internal Medicine, College of Medicine, Charles R. Drew University of Medicine and Science, 1731 E. 120th St, Los Angeles, CA 90059, USA; b Department of Family Medicine, College of Medicine, Charles R. Drew University of Medicine and Science, 1731 E. 120th St, Los Angeles, CA 90059, USA; c Integrated Substance Abuse Programs, Department of Psychiatry and Biobehavioral Sciences, University of California, Los Angeles, 10911 Weyburn Ave, Suite 200, Los Angeles, CA 90024-2886, USA; d Partnership to End Addiction, 711 Third Ave, 5th Floor, Suite 500, New York City, NY 10017, USA

**Keywords:** Stress, Adolescence, Puberty, Alcohol, Sex, Substance use

## Abstract

**Background:**

Early life stress (ELS) is associated with an increased risk of substance use in adolescence. The interaction of puberty with neurodevelopment during adolescence increases the sensitivity of the brain to both sex and stress hormones. This sensitivity may result in sex-specific pathways from ELS to adolescent alcohol use initiation. The current study examines the effect of ELS on alcohol initiation by age 13 via pubertal mediators, separated by sex and adjusted for the independent effects of age.

**Methods:**

Adolescents from the Adolescent Brain Cognitive Development study (ABCD; N = 4828 US children aged 9–10 at baseline) were assessed for ELS, age (in months), pubertal mediators (pubertal development score or PDS, testosterone) from baseline through year 2, and alcohol use initiation from baseline through year 3. Confirmatory factor analysis was used to generate a latent factor of ELS from baseline measures of threat and deprivation, which was then placed into a combined measurement and structural model to assess the mediation of ELS and age effects by pubertal measures in a sex-stratified analysis of alcohol use initiation.

**Results:**

ELS was not significantly associated with alcohol initiation in either sex. Age was directly associated with alcohol initiation among males (0.07 SE 0.02 p < 0.01) and indirectly associated among females (0.03 SE 0.01 p < 0.01). This indirect effect of age was mediated by longitudinal measures of pubertal development (PDS and testosterone). ELS was significantly associated with both PDS and testosterone at baseline in both sexes.

**Conclusions:**

Our findings highlight sex-specific effects of age on alcohol use initiation, with females showing an indirect effect of age on alcohol initiation mediated by pubertal measures, and males showing a direct effect of age. Future studies should investigate sex differences in the interactions of testosterone and PDS with social and individual factors influencing alcohol use initiation. Recognizing the different pathways influencing male and female early adolescent alcohol use initiation can help health providers and parents tailor alcohol prevention strategies to address the needs of their adolescent.

## 1. Introduction

Adolescent substance use is an important public health issue due to its potential for long-term impacts and negative health outcomes. Earlier initiation of substance use is associated with an increased risk of substance use disorder (SUD) later in life (King and Chassin, 2007). Prior research has identified risk factors that increase the likelihood of problematic adolescent substance use (Bayly and Vasilenko, 2021). For example, Villodas et al. (2021) performed a latent class analysis grouping adolescents based on dichotomous indicators of abuse and neglect. Those in the multi-type class had 7–9-fold the amount of illicit SUD symptoms than those in the low-risk class. Similarly, a Canadian study of adolescents assessed the effects of child welfare involvement (taken as an indicator of maltreatment) on Global Appraisal of Needs-Short Screener (GAINS-SS) scores across a baseline, 2-year, and 4-year follow up period. Relative to the adolescents reporting no child welfare involvement, those with involvement were more likely to belong to the high-risk relapse SUD trajectory class, defined by initially declining and then increasing symptom scores on the GAINS-SS (Zhu et al., 2023). Early life stress (ELS) encompasses many of these experiences of childhood trauma or abuse and expands beyond these factors to include socioeconomic and neighborhood stressors. These additional stressors describe a context in which the more proximal factors of family and trauma interact to generate life experiences (Fagan et al., 2015).

ELS is also associated with earlier puberty (Senger-Carpenter et al., 2024). Early puberty is defined as the development of secondary sexual characteristics significantly earlier than peers (Downing and Bellis, 2009) and is associated with emotional and behavioral problems (Vijayakumar et al., 2023). Among females, MacSweeney et al. (2025) showed that youth with a higher exposure to trauma in childhood had a more advanced pubertal status compared to their peers. Additionally, findings from the Millennium Cohort Study revealed girls from the poorest income quantile were twice as likely as girls in the richest quantile to have started menstruation by age 11 (Kelly et al., 2017). This suggests that both threat and deprivation contribute to early maturation in females.

Researchers hypothesize that earlier pubertal development results in association with an older peer group more likely to engage in substance use, resulting in earlier substance use initiation (Kim et al., 2017) especially in females (Stepanyan et al., 2020). The association between early puberty and substance use initiation is found more consistently among females than males (Granata et al., 2024; Stumper et al., 2019), suggesting sex differences in the pathways leading to early initiation of substance use. This may also be due to the increase in internalizing symptoms common among girls who start puberty before their peers (Barendse et al., 2022). Higher symptoms of anxiety and depression may lead to the adoption of substance use as a coping mechanism. Indeed, a recent study by Romano et al. (2021) found significantly more adolescent females than males reported using substances to cope with COVID-19 related changes.

A recent model (Donovan et al., 2024) outlined a potential mechanism to explain the observed sex differences, whereby ELS impacts female hormone levels more strongly than males, altering neurodevelopmental processes and increasing risk of early substance use. Although both sexes are sensitive to ELS, studies have shown females are more likely to exhibit hormonal dysregulation. A study of 16-year-olds’ response to a social stress test found that females whose parents reported a history of depressive symptoms displayed a blunted cortisol response not seen in males (Bouma et al., 2011). Among children and early adolescents, daily cortisol levels are positively coupled with testosterone, an effect which is normally stronger in females than males (Marceau et al., 2015). However, a study of hormone coupling in 9-year-old youths found cortisol and testosterone morning levels to be less tightly coupled in females with a history of stressful life events, but not in males with a similar history (Black et al., 2018). This suggests that the degree to which ELS influences testosterone may differ between males and females, even in early adolescence.

During adolescence, the brain is particularly sensitive to sex hormones (Blakemore et al., 2010), driving alterations in the connectivity between the executive control regions in the prefrontal cortex (PFC) and the reward regions of the brain (Arain et al., 2013). For example, a 2021 study of adolescents aged 12–14 reported that higher testosterone levels were associated with decreased activation of the PFC during receipt of a monetary reward (Poon et al., 2019). Additionally, adolescents and young adults (aged 10–24) in a longitudinal study showed morning testosterone levels predicted alcohol use 2 years later, with higher levels of testosterone significantly predicting a higher number of average glasses of alcohol consumed per night (Braams et al., 2016). Given these study results, the authors tested the hypothesis that testosterone mediates the impact of ELS on later alcohol use more strongly in adolescent females than in males.

The current manuscript uses a large, national longitudinal database to examine the relationships between ELS, pubertal development, testosterone levels, and their association with initiation of alcohol use while adjusting for the effects of age. The size and heterogeneity of the Adolescent Brain Cognitive Development (ABCD) database allows for examination of more subtle combinations of effects that may not reach significance in smaller homogenous study populations. Additionally, the younger baseline (ages 9–10) and frequent data collection (every 6 months) make this database particularly useful for tracking incidence of substance use. These methodological advantages over previous studies are expected to advance what we know about the mechanisms by which puberty and sex hormones carry the effects of ELS on alcohol use. The authors hypothesize that age at baseline and ELS will be significantly associated with initiating alcohol use by age 13. Given the connections between ELS, early pubertal timing and subsequent substance use, the authors hypothesize that development of secondary sexual characteristics mediates the effect of ELS on alcohol initiation, though perhaps not as strongly as testosterone. Finally, analyzing males and females separately will provide valuable insight into how each of these factors effect alcohol use initiation within biological sex, allowing for the comparison of mediating effects of pubertal development score (PDS) and testosterone.

## 2. Methods

### 2.1. Study design

Data for the present analysis comes from the Adolescent Brain Cognitive Development (ABCD) study. ABCD is a national, longitudinal study of adolescents recruited at ages 9–10 across 22 sites (Garavan et al., 2018; Volkow et al., 2018). Employing a mix of yearly in-person and mid-year phone interviews, ABCD has released multiple waves of data. The current secondary data analysis utilizes data from baseline through the third annual visit, as represented in data release 5.1; age range of the study sample at baseline (N = 4828) is 9–11 years, with an average of 9.9 (±0.02) years.

All ABCD study procedures were approved via a centralized Institutional Review Board at University of California, San Diego (Barch et al., 2018; Hoffman et al., 2019). Youth gave assent and parents/guardians gave informed consent for all procedures during each session. Post-stratification weights based on the American Community Survey were utilized during descriptive analysis to account for complex sampling within the study, as recommended in Heeringa and Berglund (2020). Participants missing data on ELS (n = 1207) or alcohol use information from more than one visit between baseline and year 3 (n = 123) were removed from the analytical sample, as were twins and triplets (n = 1860), and youth reporting any full substance use (more than a puff or sip of nicotine, marijuana, or alcohol) at baseline (n = 70). The remaining participants were assessed for validity and completeness of longitudinal testosterone and pubertal development measures spanning baseline through year 2 (n = 3780 removed), such that the final analytical sample was composed of 4828 participants with full and complete data.

### 2.2. Salivary hormone measurements

Salivary testosterone levels (reported in pg/mL in Table 1) measured at baseline, year 1, and year 2 represent an average of two repeat measures of a single sample of salivary hormones collected during the in-person interview (occurring between 0700 and 1900) via passive drool method as noted in Uban et al. (2018). Using R 4.2.3, replicate values were assessed for validity and quality control, and a mean value was established for use (Herting et al., 2020). Mean value is reported in Table 1, and regression analysis of log-transformed values were performed to adjust for confounds such as time of day, medication use, and caffeine use. Residuals were used for outcome modeling as detailed in (Barendse et al., 2024).

### 2.3. Pubertal development score

Parental report of PDS was used for the baseline measure to reduce over- or under- reporting of pubertal development by youth as recommended (Cheng et al., 2021; Herting et al., 2020). Measures for year 1 and year 2 were the average of parental and youth reports. For each question, development of secondary sexual characteristics was ranked from 1 (has not started) through 4 (seems complete). PDS was calculated as the average from three unisex and two sex-specific (two for males, two for females) questions, with no participants missing more than one answer (Herting et al., 2020). A previous study of associations between ELS and substance use initiation identified PDS at baseline as a predictor of earlier substance use initiation (Donovan et al., 2025). Its effect as a mediator is combined with that of testosterone levels to better model effects of overall pubertal development.

### 2.4. ELS as a latent factor

ELS was included in the model as a latent factor, composed of measures describing the socioeconomic, family environment, parental history, and traumatic event history of youths as measured at baseline. Briefly, ELS were assessed from reports of parents and youth. The items selected for inclusion were derived from a confirmatory factor analysis of ELS measures identified in previous ABCD studies (Brieant et al., 2023; Donovan et al., 2025; Hoffman et al., 2019; Orendain et al., 2023). Family financial issues such as inability to pay for rent, food, health care or utilities in the past 12 months were included as a count measure (Total Family Hardship, 0–7 max). The average of parent and youth ratings from the conflict subsection of the Family Environment Scale described day to day family environment over the last 12 months with higher ratings indicating increased conflict (Family Conflict, 0–9 max). The history of parental psychological problems including substance use was recorded as lifetime measures (Parental History of Mental Health/Substance Use Disorder (MH/SUD), 0–11 max). Finally, the Kiddie Schedule for Affective Disorders/Schizophrenia-Post Traumatic Stress Disorder parental survey described the number of traumatic events experienced by youth across their lifetime (KSADS Trauma 0–17 max).

### 2.5. Alcohol use measurements

Alcohol use from baseline through year 3 was defined as consumption of a full standard unit of alcohol (Sullivan et al., 2022). Consumption of less than this amount was defined as experimentation and not considered as “use” within the current study. Consumption of substances for religious purposes was not considered “use”. Thus, the measure included here is alcohol use incidence and was represented as a binary value (0 = no use, 1 = use).

### 2.6. Statistical analyses

Descriptive analyses were performed in SAS Version 9.4, using SURVEYMEANS and SURVEYFREQ procedures to accommodate the nested sample design and population weighting of the ABCD study data (Garavan et al., 2018). Means were compared across sex (Table 1), with Bonferroni adjustment for multiple comparisons and R-squared values to report effect size of significant differences. Race/ethnicity was also compared across sex via Wald Chi-Squared test to verify similar makeup of male and female samples as well as relevance to national demographics. Bivariate Pearson correlation of independent variables and mediators were calculated for continuous measures, with Spearman’s Rho reported for correlations with the binary outcome of alcohol initiation. These values are displayed by sex in Table 2.

Confirmatory factor analysis determined the best composition of measures (described in 2.4) for the latent factor ELS. Using covariance analysis of linear structural equations (CALIS), a four-item measurement model best fit both males (Root Mean Square Error of Approximation, RMSEA= 0.04, Comparative Fit Index, CFI= 1.0) and females (RMSEA= 0.0, CFI=1.0), with all standardized factor loading estimates greater than 0.3 (Table 3). This measurement model was then extended into our structural model, assessing males and females separately. The latent factor of ELS was used as a predictor of alcohol initiation, defined as a binary (0 = not initiated 1 =initiated) of alcohol initiation by year 3 (~13 years old).

Mediating effects of physical secondary sexual characteristics (PDS) and corrected testosterone levels (testosterone) were assessed longitudinally, including baseline, year 1, and year 2 measures. The longitudinal mediation analysis included the confounding independent variable of age as measured at baseline. This serves to delineate the independent contributions of the relationships among age, mediators over time, and alcohol initiation from those of ELS, mediators over time, and alcohol initiation. Covariance and errors of the model are not shown.

## 3. Results

### 3.1. Descriptive statistics and correlations

Age at baseline differed slightly between males (119.5 months) and females (118.8 months, p <0.01, R^2^=0.00) and there was no significant difference in race composition between sexes (p =0.7). Females displayed higher testosterone levels at baseline and year 1 (baseline male: 32.0 pg/mL vs female: 36.2 pg/mL, p <0.01, R^2^=0.01 and year 1 male: 37.8 pg/mL vs female: 40.8, pg/mL, p <0.01, R^2^=0.01), but this reversed in year 2 with males showing higher levels than females (52.5 pg/mL vs female: 48.4 pg/mL, p <0.01, R^2^=0.01). Pubertal development score differences between males (baseline PDS: 1.4, year 1 PDS: 1.6, year 2 PDS: 1.8) and females (baseline PDS: 1.8 p <.0.01 R^2^=0.1, year 1 PDS: 2.0 p <0.01 R^2^=0.1, year 2 PDS: 2.4 p <0.01 R^2^=0.2) increase in effect size longitudinally (Table 1). Components used in generating the latent factor ELS were not significantly different between sexes, except for Family Conflict where males displayed significantly higher rates than females (males: 2.3 vs females: 2.1 p <0.01 R^2^=0.01), but the effect size was very low. There was no significant difference between male and female alcohol initiation incidence (p =0.9).

Bivariate correlations revealed significant correlations between alcohol initiation and age for males (0.07, p < 0.01), while females displayed significant correlations between alcohol initiation and testosterone at baseline (0.05, p = 0.01) (Table 2). Both males and females displayed significant correlation between alcohol initiation and PDS at year 1 (male: 0.05, p = 0.02 vs female: 0.05, p = 0.01) and at year 2 (male and female: 0.06, p < 0.01). Additionally, most factors describing ELS (Family Hardship, Parental History of MH/SUD, KSADS Trauma) displayed significant correlations with all PDS measures in both females and males, but sex-specific correlations were also present. Family Conflict was significantly correlated with male year 1 PDS (0.04, p = 0.04), while KSADS Trauma was significantly correlated with female year 2 testosterone (0.06, p < 0.01).

### 3.2. Structural equation modeling

**Fig. 1.**
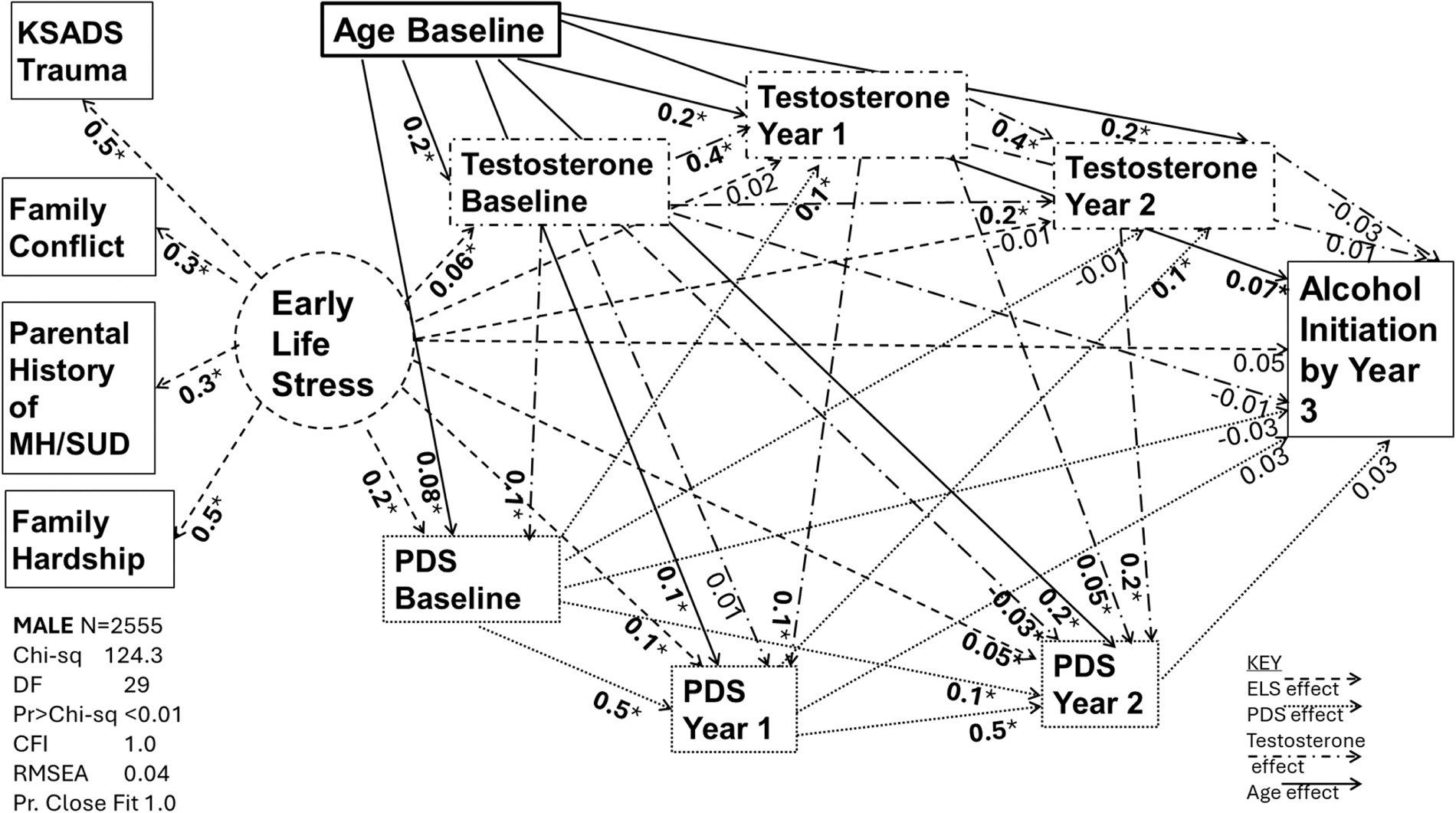
Baseline through Year 2 Testosterone and PDS as mediators of ELS and age on alcohol initiation among males, *p < 0.05.

**Fig. 2.**
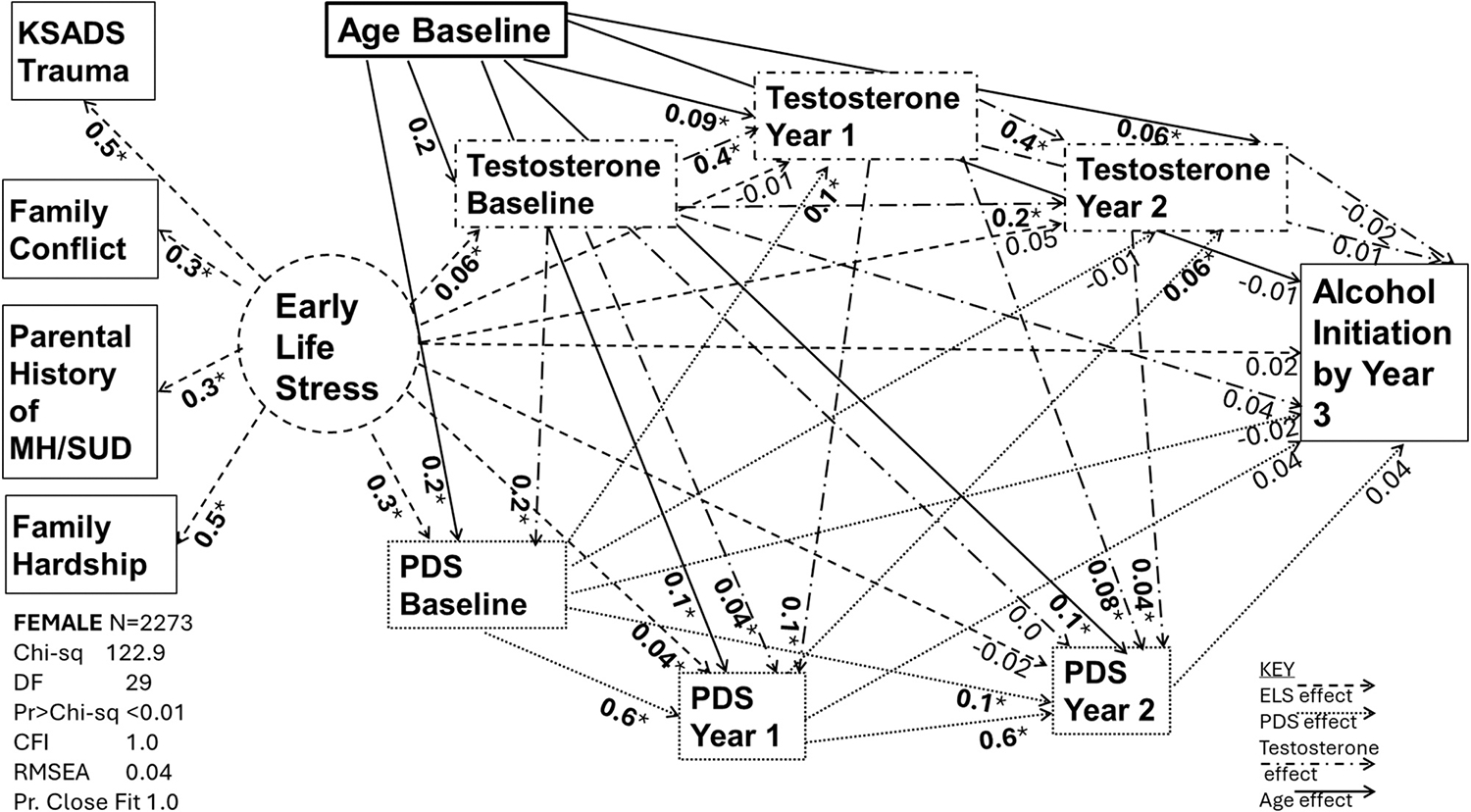
Baseline through Year 2 Testosterone and PDS as mediators of ELS and age on alcohol initiation among females, *p < 0.05.

Table 3 provides the standardized factor loadings for components used to generate the latent factor of ELS. Values were similar across sex, indicating that the composition of the latent factor works well for both males and females. Figs. 1–2 describe the path models by sex, with path coefficient values (β) reported in Table 4. Table 5 provides the total, direct, and indirect effects of the latent variable ELS on alcohol initiation for each model. Total, direct, and indirect effects of age are also included in Table 5. All values reported are standardized. Chi-sq, degrees of freedom, Comparative Fit Index, Root Mean Squared Approximation, and Probability of Close Fit are reported in each figure. Briefly, Fig. 1 displays a probability of close fit = 1.0, RMSEA= 0.04, and a CFI= 1.0, while Fig. 2 displays a probability of close fit = 1.0, RMSEA= 0.04, and a CFI= 1.0, indicating excellent fit of the model in both males and females.

The components of the latent factors contributed stably and significantly in both sexes (Table 4). Analysis of direct effects showed ELS was not associated with alcohol initiation in either females (β: 0.02 SE 0.03 p = 0.6) or males (β: 0.05 SE 0.03 p = 0.1). Age was positively associated with alcohol initiation in males (β: 0.07 SE 0.02 p < 0.01). Development of secondary sexual characteristics as measured by PDS was not significantly associated with alcohol initiation at any time in either sex (p = 0.2–0.4). Testosterone was also not significantly associated with alcohol initiation at any time in either sex (p = 0.2–0.7).

ELS was positively associated with baseline and year 1 PDS in both sexes (baseline males β: 0.2 SE 0.03 p < 0.01, year 1 males: β: 0.1 SE 0.02 p < 0.01, baseline females β: 0.3 SE 0.03 p < 0.01, year 1 females: β: 0.04 SE 0.01 p = 0.04). Age was positively associated with PDS in both sexes across time (males β: 0.08–0.2 and females β: 0.1–0.2, SE 0.01–0.02 p < 0.01). ELS was significantly associated with baseline testosterone in both males (0.06 SE 0.03 p = 0.04) and females (0.06 SE 0.03 p < 0.05), but year 1 and year 2 testosterone were not significantly associated. Age was significantly associated with baseline testosterone in both males (β: 0.2 SE 0.02 p < 0.01) and females (β: 0.2 SE 0.02 p < 0.01), and continued to be significantly associated at year 1 (male β: 0.2 SE 0.02 p < 0.01 and female β: 0.09 SE 0.02 p < 0.01) and year 2 (male β: 0.2 SE 0.02 p < 0.01 and female β: 0.06 SE 0.02 p < 0.01) in both sexes. Associations of testosterone with PDS across time varied by sex, with the largest association in males between year 2 testosterone and PDS (β: 0.2 SE 0.02 p < 0.01) and in females between baseline testosterone and PDS (β: 0.2 SE 0.02 p < 0.01). PDS associations with testosterone were strongest for males between year 1 PDS and year 2 testosterone (β: 0.1 SE 0.02 p < 0.01) and strongest for females between baseline PDS and year 1 testosterone (β: 0.1 SE 0.02 p < 0.01). No significant indirect effects were found for ELS on alcohol initiation in either sex (β: 0.0–0.01 SE 0.01, p = 0.2–0.6), but age was found to have an indirect effect on alcohol initiation among females (β: 0.03 SE 0.01 p < 0.01) (Table 5).

## 4. Discussion

### 4.1. The effects of ELS on alcohol initiation, PDS, and testosterone

ELS is known to increase the risk of SUD in adults (Felitti et al., 1998), but what is less certain is their effects on early adolescent alcohol use. Here, we present data showing no association of ELS as measured at age 9–10 with alcohol initiation by age 13 in either males or females. This is in contrast with our previous findings (Donovan et al., 2025), where higher levels of ELS were associated with an increase in instantaneous risk of alcohol use. The hypothesized mediators, PDS and testosterone, showed no association with alcohol initiation at any timepoint. The low number of adolescents initiating alcohol use (n = 73 reported having had a full drink of alcohol) may indicate that the expected relationship between ELS and alcohol initiation has yet to develop. A previous study showing substance use to be positively associated with pubertal development was in a slightly older population of adolescents (ages 11–16) (Stepanyan et al., 2020). More advanced development of secondary sexual characteristics may also increase parental monitoring or rule setting, two factors which are associated with a decrease in risk of alcohol use, especially in females (Green et al., 2024; Keogh-Clark et al., 2021; Martz et al., 2022).

ELS was associated with a significant positive effect on PDS in males, which decreased gradually from baseline through year 2. This contrasts with the pattern observed for females, where the association drops precipitously from baseline to year 1, and is not significant at year 2. This suggests an ELS-induced increase in early pubertal development among females that is not present in males, which is supported by the literature (Ho et al., 2024). Though males displayed a decreasing association over time between ELS and PDS, the pattern was more gradual and ELS remained significantly associated with PDS through year 2. ELS was also significantly associated with testosterone at baseline in both sexes, with no association for year 1 or year 2 measures, indicating a diminishing impact of ELS over time. Similarly, testosterone itself was most strongly associated with same-year PDS measures, peaking for females at baseline and males at year 2. Given the strength of the contemporaneous measures of ELS and pubertal mediators, it may be that inclusion of year 1 and year 2 ELS measures would better define the influence of ELS on alcohol initiation as mediated by pubertal measures.

### 4.2. Age effects

Given the low incidence of alcohol initiation in this population (1.7 %), the significant association of age at baseline with alcohol initiation among males is notable. Age was also positively associated with PDS and testosterone, displaying sex-specific patterns. In males, the strength of the association between age and PDS and age and testosterone increased over time, with year 2 showing the strongest associations. In females, the strength of the associations between age and PDS and age and testosterone decreased from baseline to year 2. This is similar to the sex-specific patterns observed for ELS associations, suggesting that the difference in pubertal development patterns is attributed to the influence of both ELS and age at baseline. This highlights the value in analyzing age as an independent variable within the model, as the ELS effects on pubertal development exist independent of age effects. Additionally, females displayed an indirect effect of age on alcohol initiation, which was mediated by PDS and testosterone measures longitudinally from baseline to year 2.

### 4.3. Implications

Our findings indicate an impact of ELS on pubertal measures, but not on alcohol initiation. This is in line with a systematic review of prevention programming efficacy among early adolescents (grades 6 and 7, ages 11–13), which found educating combined high- and low-risk populations together to be as effective as isolating high-risk early adolescents for targeted intervention (Onrust et al., 2016). Instead, age showed direct effects in males and indirect effect in females which was mediated by pubertal measures. This finding suggests that, while parents may focus on their child’s age as an indicator of when to discuss alcohol use, this approach may be more effective for males than females. Parents and clinicians may want to consider age and pubertal status in determining when to talk to female adolescents about the risks of alcohol use.

### 4.4. Limitations

The current study is not without limitations. Our outcome measure displayed very low rates of alcohol use (1.7 %) by age 13. While any use at this age is detrimental, it is difficult to separate use types so early in the alcohol use history. Participants reporting a full drink of alcohol may develop a pattern of use as a coping mechanism beyond age 13, but no participants had reported regular use in the data used in this study. Future data releases providing information up through age 16 will be needed to better discern between experimental use and onset of chronic use.

Other social factors previously identified as predictors of adolescent substance use, such as availability of alcohol, peer attitudes toward alcohol use, or parental rules regarding alcohol use (Martz et al., 2022) may also link ELS and alcohol initiation. One component of the latent factor of ELS is parental history of mental health or substance use disorders, which may contribute to previously observed ELS-alcohol initiation associations via genetic factors (Kuo et al., 2023). Additionally, the measures comprising the latent factor of ELS used in this study were limited to those reported at baseline. It may be that ELS occurring more proximally to alcohol initiation, such as in years 1 or 2, has a stronger impact. These questions are beyond the scope of the current study, but future studies should incorporate these and other peer and parental factors to gain a better understanding of the interaction between biological and social contexts surrounding alcohol initiation, and how these may differ across sex.

### 4.5. Conclusion

The current study yields insight into the complexity of factors driving adolescent alcohol initiation. Our results showed that age is indirectly associated with alcohol initiation in females. This effect is mediated by longitudinal PDS and testosterone measures. In contrast, males displayed a direct effect of age on alcohol initiation. Though not found to be statistically significant for alcohol initiation, ELS may contribute to other measures of alcohol use, or alcohol use patterns, among older adolescent populations. These relationships should continue to be evaluated as adolescents in the ABCD study age, and alcohol becomes more prevalent.

## Figures and Tables

**Table 1:** Demographic information of included study population separated by sex, adjusted for complex samples, and weighted.

Population demographics Age 9–10 years at baseline	All participants (N = 4828)	Male (n = 2555, 53 %)	Female (n = 2273, 47 %)	p value (*t*-test/R^2^ or chi-sq, m vs f)

Age in months (mean, SE)	119.2 (0.2)	119.5 (0.3)	118.8 (0.2)	**< 0.01/0.00**
Race/Ethnicity (n, %)				0.7
Black	485 (8.7 %)	254 (8.6 %)	231 (8.9 %)	
Hispanic	973 (23.5 %)	512 (23.6 %)	461 (23.4 %)	
Other	620 (11.0 %)	311 (10.4 %)	306 (11.6 %)	
White	2750 (56.8 %)	1478 (57.5 %)	1272 (56.1 %)	
Pubertal Development Score (PDS)(mean, SE)	1.6 (0.02)	1.4 (0.02)	1.8 (0.03)	**< 0.01/0.1**
Year 1 PDS	1.8 (0.03)	1.6 (0.02)	2.0 (0.03)	**< 0.01/0.1**
Year 2 PDS	2.1 (0.02)	1.8 (0.02)	2.4 (0.03)	**< 0.01/0.2**
Testosterone (uncorrected, pg/mL) (mean, SE)	34.0 (1.2)	32.0 (1.4)	36.2 (1.1)	**< 0.01/0.01**
Year 1 Testosterone	39.3 (0.9)	37.8 (1.1)	40.8 (0.9)	**< 0.01/0.01**
Year 2 Testosterone	50.6 (1.2)	52.5 (1.3)	48.4 (1.4)	**< 0.01/0.01**
Alcohol initiation (N, %)	73 (1.7 %)	39 (1.7 %)	34 (1.8 %)	0.90
ELS measures:				
Total Family Hardship	0.5 (0.07)	0.5 (0.07)	0.5 (0.08)	0.7
Parental History of MH/SUD	1.6 (0.1)	1.5 (0.1)	1.6 (0.1)	0.4
Family Conflict	2.2 (0.06)	2.3 (0.06)	2.1 (0.07)	**< 0.01/0.00**
KSADS Trauma	0.5 (0.04)	0.5 (0.04)	0.6 (0.04)	0.3

Age in months, race/ethnicity, Pubertal Development Score, testosterone in pg/mL, alcohol use initiation, and ELS component scores. Significance adjusted for multiple comparisons (Bonferroni), bold p-value indicates significant difference between sexes.

**Table 2:** Pearson correlation values by sex for variables as measured at baseline with PDS and testosterone (corrected) at baseline, Year1, and Year 2.

		1. Total Family Hardship	2. Parent history of MH/SUD	3. Family Conflict	4. KSADS Trauma	5. Age at baseline	6. PDS	7. PDS Year 1	8. PDS Year 2	9. Testosterone	10. Testosterone Year 1	11. Testosterone Year 2	12. Alcohol initiation
		Male Pearson Correlations n = 2555											

1. Total Family Hardship	Female Pearson Correlations n = 2273	—	**0.2 p < 0.01**	**0.2 p < 0.01**	**0.1 p < 0.01**	− 0.02 p = 0.3	**0.1 p < 0.01**	**0.1 p < 0.01**	**0.09 p < 0.01**	**0.04 p = 0.04**	**0.06 p < 0.01**	**0.04 p = 0.03**	0.00 p = 0.9
2. Parent History of MH/SUD		**0.2 p < 0.01**	—	**0.1 p < 0.01**	**0.2 p < 0.01**	0.01 p = 0.7	**0.05 p < 0.01**	**0.07 p < 0.01**	**0.08 p < 0.01**	− 0.01 p = 0.6	0.00 p = 0.9	0.01 p = 0.7	0.03 p = 0.1
3. Family Conflict		**0.1 p < 0.01**	**0.2 p < 0.01**	—	**0.1 p < 0.01**	**−0.04 p = 0.04**	0.03 p = 0.1	**0.04 p = 0.04**	0.00 p = 0.9	0.00 p = 0.8	0.01 p = 0.6	− 0.04 p = 0.06	0.03 p = 0.1
4. KSADS Trauma		**0.2 p < 0.01**	**0.2 p < 0.01**	**0.1 p < 0.01**	—	0.01 p = 0.5	**0.1 p < 0.01**	**0.1 p < 0.01**	**0.1 p < 0.01**	0.03 p = 0.08	0.01 p = 0.5	0.03 p = 0.08	− 0.03 p = 0.1
5. Age at baseline		0.00 p = 0.8	− 0.03 p = 0.2	− 0.03 p = 0.1	− 0.03 p = 0.9	—	**0.09 p < 0.01**	**0.2 p < 0.01**	**0.4 p < 0.01**	**0.2 p < 0.01**	**0.2 p < 0.01**	**0.3 p < 0.01**	**0.07 p < 0.01**
6. Pubertal development (PDS)		**0.2 p < 0.01**	**0.1 p < 0.01**	0.03 p = 0.1	**0.1 p < 0.01**	**0.3 p < 0.01**	—	**0.5 p < 0.01**	**0.4 p < 0.01**	**0.1 p < 0.01**	**0.2 p < 0.01**	**0.2 p < 0.01**	0.01 p = 0.7
7. PDS Year 1		**0.1 p < 0.01**	**0.1 p < 0.01**	0.03 p = 0.2	**0.08 p < 0.01**	**0.3 p < 0.01**	**0.7 p < 0.01**	—	**0.7 p < 0.01**	**0.2 p < 0.01**	**0.3 p < 0.01**	**0.3 p < 0.01**	**0.05 p = 0.02**
8. PDS Year 2		**0.1 p < 0.01**	**0.07 p < 0.01**	0.00 p = 1.0	**0.06 p < 0.01**	**0.4 p < 0.01**	**0.6 p < 0.01**	**0.8 p < 0.01**	—	**0.2 p < 0.01**	**0.4 p < 0.01**	**0.5 p < 0.01**	**0.06 p < 0.01**
9. Testosterone		**0.09 p < 0.01**	− 0.04 p = 0.08	− 0.01 p = 0.7	0.01 p = 0.6	**0.2 p < 0.01**	**0.3 p < 0.01**	**0.3 p < 0.01**	**0.3 p < 0.01**	—	**0.5 p < 0.01**	**0.4 p < 0.01**	− 0.01 p = 0.7
10. Testosterone Year 1		**0.06 p < 0.01**	0.02 p = 0.4	− 0.01 p = 0.7	0.01 p = 0.6	**0.2 p < 0.01**	**0.3 p < 0.01**	**0.3 p < 0.01**	**0.4 p < 0.01**	**0.5 p < 0.01**	—	**0.5 p < 0.01**	0.01 p = 0.5
11. Testosterone Year 2		**0.07 p < 0.01**	− 0.01 p = 0.7	− 0.00 p = 0.9	**0.06 p < 0.01**	**0.2 p < 0.01**	**0.2 p < 0.01**	**0.3 p < 0.01**	**0.3 p < 0.01**	**0.4 p < 0.01**	**0.5 p < 0.01**	—	0.01 p = 0.7
12. Alcohol initiation		0.01 p = 0.5	0.03 p = 0.1	0.01 p = 0.7	0.00 p = 0.9	0.01 p = 0.5	0.03 p = 0.2	**0.05 p = 0.01**	**0.06 p < 0.01**	**0.05 p = 0.01**	0.04 p = 0.05	0.02 p = 0.4	—

**Table 3:** Standardized factor loadings for the latent factor of ELS used in outcome analyses.

Standardized Factor Loading Matrix (all p < 0.01) Males N = 2555	Males		Females	
Females N = 2273	Estimate Standard Error		Estimate Standard Error	

Total Family Hardship	0.4	0.03	0.4	0.03
Parental History of MH/SUD	0.5	0.03	0.5	0.03
Family Conflict	0.3	0.03	0.3	0.03
KSADS Trauma	0.4	0.03	0.4	0.03

Estimate and standard error, all values p < 0.01.

**Table 4:** Standardized estimates of path coefficients.

Standardized Estimate of Path Coefficients β (SE) p-value	Male	Female

Family Hardship→ELS	**0.5 (0.01) p < 0.01**	**0.5 (0.02) p < 0.01**
Parental History→ELS	**0.3 (0.01) p < 0.01**	**0.3 (0.01) p < 0.01**
Family Conflict→ELS	**0.3 (0.01) p < 0.01**	**0.3 (0.01) p < 0.01**
KSADS Trauma→ELS	**0.5 (0.01) p < 0.01**	**0.5 (0.02) p < 0.01**
ELS→Alcohol Initiation	0.05 (0.03) p = 0.1	0.02 (0.03) p = 0.6
Age→Alcohol Initiation	**0.07 (0.02) p < 0.01**	− 0.01 (0.02) p = 0.6
PDS→Alcohol Initiation	− 0.03 (0.02) p = 0.2	− 0.02 (0.03) p = 0.4
PDS Year 1 → Alcohol Initiation	0.03 (0.03) p = 0.3	0.04 (0.04) p = 0.3
PDS Year 2 → Alcohol Initiation	0.03 (0.03) p = 0.3	0.04 (0.03) p = 0.2
Testosterone→ Alcohol Initiation	− 0.01 (0.02) p = 0.6	0.04 (0.02) p = 0.2
Testosterone Year 1→ Alcohol Initiation	0.01 (0.02) p = 0.6	0.01 (0.03) p = 0.7
Testosterone Year 2→ Alcohol Initiation	− 0.03 (0.03) p = 0.2	− 0.02 (0.03) p = 0.4
ELS→PDS	**0.2 (0.03) p < 0.01**	**0.3 (0.03) p < 0.01**
Age→PDS	**0.08 (0.02) p < 0.01**	**0.2 (0.02) p < 0.01**
Testosterone→PDS	**0.1 (0.02) p < 0.01**	**0.2 (0.02) p < 0.01**
ELS→PDS Year 1	**0.1 (0.02) p < 0.01**	**0.04 (0.02) p = 0.04**
Age→PDS Year 1	**0.1 (0.02) p < 0.01**	**0.1 (0.01) p < 0.01**
PDS→PDS Year 1	**0.5 (0.02) p < 0.01**	**0.6 (0.01) p < 0.01**
Testosterone→ PDS Year 1	0.01 (0.02) p = 0.4	**0.04 (0.02) p = 0.02**
Testosterone Year 1 → PDS Year 1	**0.1 (0.02) p < 0.01**	**0.1 (0.02) p < 0.01**
ELS→PDS Year 2	**0.05 (0.01) p = 0.02**	− 0.02 (0.02) p = 0.3
Age→PDS Year 2	**0.2 (0.01) p < 0.01**	**0.1 (0.01) p < 0.01**
PDS→PDS Year 2	**0.1 (0.02) p < 0.01**	**0.1 (0.02) p < 0.01**
PDS Year 1→PDS Year 2	**0.5 (0.02) p < 0.01**	**0.6 (0.02) p < 0.01**
Testosterone→ PDS Year 2	**−0.03 (0.01) p = 0.03**	− 0.00 (0.01) p = 0.9
Testosterone Year 1 → PDS Year 2	**0.05 (0.02) p < 0.01**	**0.08 (0.02) p < 0.01**
Testosterone Year 2 → PDS Year 2	**0.2 (0.02) p < 0.01**	**0.04 (0.02) p < 0.01**
ELS→ Testosterone	**0.06 (0.03) p = 0.04**	**0.06 (0.03) p < 0.05**
Age→ Testosterone	**0.2 (0.02) p < 0.01**	**0.2 (0.02) p < 0.01**
ELS→ Testosterone Year 1	0.02 (0.03) p = 0.5	− 0.01 (0.03) p = 0.7
Age→ Testosterone Year 1	**0.2 (0.02) p < 0.01**	**0.09 (0.02) p < 0.01**
PDS→ Testosterone Year 1	**0.1 (0.02) p < 0.01**	**0.1 (0.02) p < 0.01**
Testosterone→ Testosterone Year 1	**0.4 (0.01) p < 0.01**	**0.4 (0.02) p < 0.01**
ELS→ Testosterone Year 2	− 0.01 (0.02) p = 0.6	0.05 (0.03) p = 0.08
Age→ Testosterone Year 2	**0.2 (0.02) p < 0.01**	**0.06 (0.02) p < 0.01**
PDS→ Testosterone Year 2	− 0.01 (0.02) p = 0.7	− 0.01 (0.03) p = 0.7
PDS Year 1 → Testosterone Year 2	**0.1 (0.02) p < 0.01**	**0.06 (0.03) p = 0.01**
Testosterone→ Testosterone Year 2	**0.2 (0.02) p < 0.01**	**0.2 (0.02) p < 0.01**
Testosterone Year 1 → Testosterone Year 2	**0.4 (0.02) p < 0.01**	**0.4 (0.02) p < 0.01**

**Table 5:** Standardized total, direct, and indirect effects with standard errors, bold indicates p-values < 0.05.

Standardized Total, Direct, and Indirect Effects on Alcohol Initiation Effect (SE) p-value	Males			Females		
	Total	Direct	Indirect	Total	Direct	Indirect

**ELS** (Baseline thru Year 2 mediators)	0.05 (0.03) p = 0.09	0.05 (0.03) p = 0.1	0.0 (0.01) p = 0.6	0.03 (0.03) p = 0.4	0.02 (0.03) p = 0.6	0.01 (0.01) p = 0.2
**Age** (Baseline thru Year 2 mediators)	**0.07 (0.02) p < 0.01**	**0.07 (0.02) p < 0.01**	0.0 (0.01) p = 0.6	0.01 (0.02) p = 0.5	− 0.01 (0.02) p = 0.6	**0.03 (0.01) p < 0.01**

Baseline through Year 2 PDS and testosterone measures as mediators.
